# Effects of phytosomal curcumin on anthropometric parameters, insulin resistance, cortisolemia and non-alcoholic fatty liver disease indices: a double-blind, placebo-controlled clinical trial

**DOI:** 10.1007/s00394-019-01916-7

**Published:** 2019-02-22

**Authors:** Arrigo F. G. Cicero, Amirhossein Sahebkar, Federica Fogacci, Marilisa Bove, Marina Giovannini, Claudio Borghi

**Affiliations:** 1 Atherosclerosis Research Unit, Medical and Surgical Sciences Department, Sant’Orsola-Malpighi Hospital, University of Bologna, Via Albertoni, 15, 40138 Bologna, Italy; 2 Biotechnology Research Center, Pharmaceutical Technology Institute, Mashhad University of Medical Sciences, Mashhad, Iran; 3 Neurogenic Inflammation Research Center, Mashhad University of Medical Sciences, Mashhad, Iran

**Keywords:** Curcumin, Phosphatidylserine, NAFLD, Diabetes, Metabolic syndrome, Clinical trial

## Abstract

**Purpose:**

Curcumin has shown to exert a positive impact on human glucose metabolism, even if its bioavailability is usually very low. The present study aimed to explore the effect of phosphatidylserine- and piperine-containing curcumin phytosomes on a large number of metabolic parameters related to insulin resistance, in the context of a randomized double-blind placebo-controlled trial involving 80 overweight subjects with suboptimal fasting plasma glucose.

**Methods:**

Subjects were randomized to be treated with indistinguishable tablets (2 per day, to be taken after dinner) containing 800 mg phytosomal curcumin (Curserin®: 200 mg curcumin, 120 mg phosphatidylserine, 480 mg phosphatidylcholine and 8 mg piperine from *Piper nigrum* L. dry extract) for 8 weeks.

**Results:**

After 56-day treatment, the curcumin-treated group experienced a significant improvement in fasting plasma insulin (FPI), HOMA index, waist circumference, blood pressure, triglycerides (TG), HDL-C, liver transaminases, gamma-GT, index of liver steatosis and serum cortisol compared to the baseline. FPI, TG, liver transaminases, fatty liver index and serum cortisol level also significantly improved compared with the placebo-treated group. Compared to the baseline, at the end of the study placebo group experienced an improvement only in FPG and TG.

**Conclusion:**

In conclusion, the present trial shows that supplementation with a phytosomal preparation of curcumin containing phosphatidylserine and piperine could improve glycemic factors, hepatic function and serum cortisol levels in subjects with overweight and impaired fasting glucose.

## Introduction

Overweight and obesity, in particular in the form of abdominal adiposity, are known to be closely associated with increased morbidity and mortality due to cardiovascular disease [1]. Several lines of evidence have revealed the interrelation between overweight and obesity and a number of cardiometabolic disorders including type 2 diabetes, dyslipidemia and non-alcoholic fatty liver disease (NAFLD) [2]. Therefore, control of glucose and lipid abnormalities in overweight and obese individuals is integral to prevent cardiovascular disease development.

Nutraceuticals have recently emerged as promising candidates for the amelioration of cardiometabolic diseases. Notably, several plant-derived compounds have been shown to be efficacious in managing type 2 diabetes [3], dyslipidemia [4] and non-alcoholic fatty liver disease (NAFLD) [5]. Among these nutraceuticals, curcumin is of particular importance owing to its anti-diabetic [6], anti-lipid-lowering [7] and hepatoprotective [8] actions. Curcumin can attenuate insulin resistance which is the common pathological mechanism for obesity, type 2 diabetes, metabolic syndrome and NAFLD [9]. From a molecular standpoint, curcumin exerts its insulin-sensitizing actions through activation of insulin receptors, increasing insulin-independent glucose uptake by pancreatic β cells, increasing lipoprotein lipase activity, anti-inflammatory effects in adipose tissue and enhancement of adipokines [10].

Despite the above-mentioned benefits, maximal efficacy of curcumin in clinical practice has been challenged by the low oral bioavailability owing to limited intestinal absorption and rapid metabolism [11]. Complexation of curcumin with phospholipids in the form of phytosomes has been introduced as an effective strategy to boost curcumin’s bioavailability through improvement of intestinal absorption and metabolic stability of this phytochemical [12]. Phytosomes are complexes of phytochemicals and phospholipids that are prepared by mixing both components in an aprotic solvent, followed by solvent evaporation [13]. The amphipathic nature of phospholipids makes phytosomal phytochemical more dispersible in the aqueous media (gut and fecal medium) compared with the phytochemical alone, improving their bioavailability. Besides increased solubility and dispersibility, phytosomal systems also achieve protection of the incorporated phytochemical from degradation in the digestive tract [13]. Among the phospholipids includable in the curcumin phytosomal delivery system, phosphatidylserine seems to have anti-atherosclerotic properties [14] and to strengthen the anti-inflammatory effects of curcumin [15]. Piperine could also improving curcumin bioavailability reducing liver conjugation of curcumin with glucuronic acid and consequently its elimination by urine [16, 17].

In a recent pilot study, the value of PS- and piperine-containing curcumin phytosomes in improving weight management in overweight subjects was reported [18]. However, possible effects of this delivery system on glycemic, lipid and hepatic indices remain unknown. The present study aimed to explore this potential effect of curcumin phytosomes in the context of a randomized double-blind placebo-controlled trial in overweight subjects with suboptimal FPG.

## Methods

This randomized, double-blind, parallel-group, placebo-controlled clinical trial involved 80 pharmacologically untreated overweight subjects with suboptimal values of FPG, consecutively enrolled in the ambulatory service of cardiovascular disease prevention in the Medical and Surgical Sciences Department of the University of Bologna.

Inclusion criteria were age between 18 and 70 years, body mass index (BMI) between 25 and 30 kg/m^2^, and FPG levels between 100 and 125 mg/dL as confirmed in at least two sequential checks before signing the consent form.

Exclusion criteria were:

- Personal history of cardiovascular disease or coronary heart disease risk equivalent.
- Consumption of glucose-lowering drugs (oral antidiabetics, insulins) or lipid-lowering drugs (statins, fibrates, ezetimibe, omega-3 polyunsaturated fatty acids) or drugs affecting lipid metabolism (i.e., full-dosed thiazides, corticosteroids, immunosuppressants).
- Known thyroid, liver, renal or muscle diseases.

The study was fully conducted in accordance with the Declaration of Helsinki, its protocol was approved by the Ethical Committee of the University of Bologna, and informed consent was obtained from all patients before the inclusion in the study.

At baseline, patients were given standard behavioral and qualitative (not quantitative) dietary suggestions to correct unhealthy habits. Standard diet advice was given by a dietitian and/or specialist doctor. Dietitian and/or specialist doctor periodically provided instruction on dietary intake recording procedures as part of a behavior modification program and then later used the subject’s food diaries for counseling. In particular, subjects were instructed to follow general indication of a Mediterranean diet, avoiding excessive intake of dairy products and red meat-derived products during the study, maintaining overall constant dietary habits. Individuals were also encouraged to increase their physical activity by walking briskly for 20–30 min, 3–5 times per week, or by cycling. The dietary habits were monitored during the whole study duration by a semi-quantitative validated 7-day questionnaire [19].

### Treatments

After 2 weeks of diet and physical activity, patients were allocated to treatment with indistinguishable tablets (2 per day, to be taken after dinner) containing 800 mg phytosomal curcumin (Curserin®: 200 mg curcumin, 120 mg phosphatidylserine, 480 mg phosphatidylcholine, associated to 8 mg piperine from *Piper nigrum* L. dry extract). The dose was established according to a previous paper where the same curcumin–phytosome product was already tested as effective and well-tolerable [18].

The bioactives are declared to be standardized by their manufacturers (for curcumin–phytosome: Indena, Italy; for piperine Sabinsa Corporation, India). With regard to the content of bioactives inside the tablets, the standardized process is guaranteed by the analytical method performed by the producer of the finished product (SIIT, Italy).

The treatment has then continued for 8 weeks. Clinical and laboratory data were obtained at the baseline, after 4 weeks and at the end of the trial. Randomization was performed using a drawing of envelopes containing randomization codes prepared by an independent statistician and specific software. The envelopes were then further mixed and distributed to the investigators who assigned the randomization code in a progressive way to the enrolled subjects. A copy of the code was provided only to the person responsible of performing the statistical analysis.

Throughout the study, patients were instructed to take the new product first dose on the day after they were given the study product in a blinded box. At the same time, all unused products were retrieved for inventory. Product compliance was assessed by counting the number of product doses returned at the time of specified clinic visits. At the end of the study, patients were asked to evaluate the treatment acceptability in term of “very low”, “low”, “good”, or “very good”.

### Assessments

All plasma parameters were obtained after a 12-h overnight fast. Venous blood samples were drawn by a nurse from all patients between 8:00 a.m. and 9:00 a.m. Plasma used was obtained by addition of Na_2_EDTA (1 mg = mL) and centrifuged at 3000*g* for 15 min at ambient temperature. Immediately after centrifugation, plasma samples were frozen and stored at − 80 °C for no more than 3 months. The following parameters were evaluated via standardized methods: total cholesterol (TC), high-density lipoprotein-cholesterol (HDL-C), triglycerides (TG), LDL-Cholesterol (LDL-C), fasting plasma glucose (FPG), glutamic-oxalacetic transaminase (GOT), glutamic-pyruvic transaminase (GPT), and gamma-glutamyl transferase (gamma-GT) [20].

Fasting plasma insulin (FPI) was assessed by enzyme linked immunoassay kits (DiaMetra, Milano, Italy). The intra- and interassay CVs for insulin were 3.1 and 6.2%, respectively. Total serum cortisol was measured by immunoassay (Calbiotech, Spring Valley, USA).

Lipid accumulation product (LAP) was calculated as (waist circumference − 65) × TG (expressed in mmol/L) for men and (waist circumference − 58) × TG (expressed in mmol/L) for women [21], and hepatic steatosis index (HSI) was calculated from 8 × GPT/GOT ratio + body mass index (+ 2 for women) [22].

Fatty liver index (FLI) was calculated as follows: [e^0.953 × ln (TG) + 0.139 × BMI + 0.718 × ln (GGT) + 0.053 × WC − 15.745^/(1 + e^0.953 × ln (TG) + 0.139 × BMI + 0.718 × ln (GGT) + 0.053 × WC − 15.745^)] × 100 [23].

All measurements were performed by trained personnel in the Lipid Clinic laboratory of the Medicine and Surgery Sciences Department, S. Orsola-Malpighi University Hospital.

### Statistical analysis

Data have been analyzed using intention to treat by means of the Statistical Package for Social Science (SPSS) 21.0, version for Windows. The sample size suggested to detect a mean difference of 10% between treatments in term of HOMA-IR reduction, with a power of 0.90 and an alpha error of 0.05, was of at least 35 subjects per group. As per protocol, we decided a priori to check the efficacy of treatments in subjects assuming at least 90% of the tested products doses foreseen by the trial design. Normally distributed baseline characteristics of the population have been compared using Student’s *t* test and *χ*^2^ test followed by Fisher’s exact test for categorical variables. Between group difference was assessed by repeated-measures ANOVA followed by the Tukey’s post hoc test. All data are expressed as means and SD. A *p* level of < 0.05 was considered significant for all tests.

## Results

All patients completed the trial and no patient experienced any adverse event. Three subjects claimed abdominal discomfort in the curcumin-treated group. Compliance was high, being 92% in both treatment groups. The overall acceptability of the proposed treatment was defined as good–very good by 90% of the curcumin-treated patients and by 86% of the placebo-treated ones.

At the baseline, enrolled subjects had similar age (54 ± 3 vs. 53 ± 5 years old, *p* > 0.05) and gender distribution (22/40 vs. 21/40 women, *p* > 0.05) between groups, and also the baseline clinical features and laboratory analyses were similar (Tables 1, 2, 3).

**Table 1:** Anthropometric and hemodynamic characteristics of the enrolled subjects at the baseline and during treatment

	Phytosomal curcumin (*n*. 40)			Placebo (*n*. 40)		
	T0	T1 (28 days)	T2 (56 days)	T0	T1 (28 days)	T2 (56 days)
BMI (kg/m^2^)	27.1 ± 1.8	26.8 ± 1.7	26.3 ± 1.4*	26.9 ± 1.9	26.5 ± 1.6	26.4 ± 1.7
WC (cm)	94 ± 7	93 ± 6	89 ± 4*	93 ± 8	92 ± 8	90 ± 9
SBP (mmHg)	131 ± 8	128 ± 9	126 ± 6*	129 ± 9	127 ± 10	127 ± 9
DBP (mmHg)	84 ± 6	85 ± 7	84 ± 7	83 ± 7	82 ± 8	81 ± 6

*BMI* body mass index, *WC* waist circumference, *SBP* systolic blood pressure, *DBP* diastolic blood pressure

*p < 0.05 vs. baseline

**Table 2:** Metabolic characteristics of the enrolled subjects at the baseline and during treatment

	Phytosomal curcumin (*n*. 40)			Placebo (*n*. 40)		
	T0	T1 (28 days)	T2 (56 days)	T0	T1 (28 days)	T2 (56 days)
FPG (mg/dL)	108 ± 9	104 ± 7	101 ± 6*	110 ± 10	107 ± 9	105 ± 8*
FPI (mcU/mL)	18 ± 4	15 ± 4*	15 ± 3*°	19 ± 5	18 ± 6	18 ± 5
HOMA-IR	4.9 ± 1.1	3.9 ± 1.2*°	3.8 ± 1.1*°	5.2 ± 1.3	4.7 ± 1.3	4.7 ± 1.4
TC (mg/dL)	193 ± 15	188 ± 12	185 ± 13	195 ± 16	190 ± 17	189 ± 19
TG (mg/dL)	185 ± 21	169 ± 18	151 ± 16*°	181 ± 22	176 ± 18	157 ± 19*
HDL-C (mg/dL)	40 ± 3	42 ± 3	44 ± 4*	41 ± 4	42 ± 4	42 ± 3
LDL-C (mg/dL)	116 ± 11	108 ± 9	111 ± 8	118 ± 12	112 ± 13	116 ± 14
Non LDL-C (mg/dL)	145 ± 13	127 ± 14*	107 ± 13*°	140 ± 15	134 ± 15	115 ± 16*

*FPG* fasting plasma glucose, *FPI* fasting plasma insulin, *HOMA-IR* homeostasis insulin resistance index, *TC* total cholesterol, *TG* triglycerides, *HDL-C* high-density lipoprotein cholesterol, *LDL-C* low density lipoprotein cholesterol

*p < 0.05 vs. baseline; °*p* < 0.05 vs. placebo

**Table 3:** Liver parameters of the enrolled subjects at the baseline and during treatment

	Phytosomal curcumin (N. 40)			Placebo (N. 40)		
	T0	T1 (28 days)	T2 (56 days)	T0	T1 (28 days)	T2 (56 days)
GOT (U/L)	21 ± 6	18 ± 3	16 ± 5*°	23 ± 5	21 ± 7	21 ± 8
GPT (U/L)	23 ± 7	21 ± 3	15 ± 3*°	24 ± 7	22 ± 6	21 ± 9
gGT (U/L)	28 ± 9	25 ± 7	24 ± 6*	27 ± 9	26 ± 9	26 ± 7
LAP	64 ± 14	63 ± 12	62 ± 10*	65 ± 13	64 ± 12	64 ± 13
HSI	38 ± 5	36 ± 5	35 ± 4*	39 ± 6	38 ± 5	37 ± 4
FLI	57 ± 11	55 ± 9	54 ± 9*°	58 ± 10	57 ± 9	57 ± 10

*GOT* glutamic oxaloacetic transaminase, *GPT* glutamate pyruvate transaminase, *gGT* gamma-glutamyl transferase, *LAP* lipid accumulation product, *HSI* hepatic steatosis index, *FLI* fatty liver index

*p < 0.05 vs. baseline; °*p* < 0.05 vs. placebo

From the randomization visit to the end of the study, the enrolled subjects maintained overall a similar dietary pattern, without significant change in total energy, total cholesterol and total saturated fatty acid intake.

**Fig. 1:**
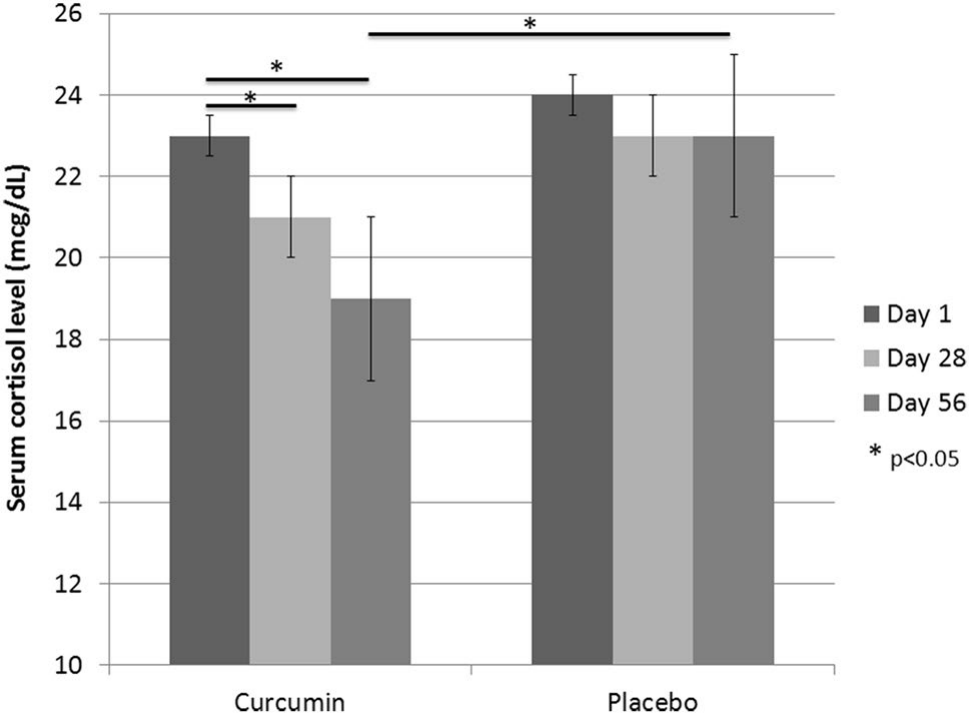
Serum cortisol level of the enrolled subjects at the baseline and during treatment

Compared to the baseline, the curcumin-treated subjects experienced a statistically significant improvement in FPG, FPI, HOMA index and serum cortisol (Table 2; Fig. 1) level after 28 days (*p* < 0.05). The improvement in HOMA index was also significant when compared to the placebo-treated group. After 56 days, the curcumin-treated group experienced a significant improvement in body mass index (− 0.8 ± 0.2 kg/m^2^), waist circumference (− 5 ± 2 cm), systolic blood pressure (− 5 ± 3 mmHg), TG (− 34 ± 18 mg/dL), HDL-C (+ 4 ± 2 mg/dL), FPG (− 7 ± 3 mg/dL), FPI (− 3 ± 2 mcU/mL), HOMA-IR (− 1.1 ± 0.6), GOT (− 5 ± 2 U/L), GPT (− 8 ± 3 U/L), gamma-GT (− 4 ± 1 U/L), LAP (− 2 ± 1), and HSI (− 3 ± 1) compared to the baseline (*p* < 0.05). FPI, TG, GOT, GPT, FLI and serum cortisol level also significantly improved compared with the placebo-treated group (*p* < 0.05) (Tables 1, 2, 3).

Compared to the baseline, at the end of the study placebo group experienced an improvement in FPG and TG (both, *p* < 0.05), only (Table 2). This effect was expected based on the lifestyle improvement prescribed in both treatment groups.

## Discussion

Findings of the present study revealed a significant improvement in glycemic and hepatic function indices along with serum cortisol concentrations following supplementation with a phytosomal preparation of curcumin in overweight subjects with suboptimal FPG. These findings have an important implication for the management of patients with overweight and obesity, metabolic syndrome and NAFLD, all of which being public health concerns worldwide. The present findings on the improvement of glycemic indices by curcumin are consistent with those of a previous investigation administering curcumin (1000 mg/day) plus piperine (10 mg/day; an absorption-enhancing alkaloid) to diabetic patients for a period of 3 months [6], where significant reductions in serum levels of glucose, HbA1c, C-peptide, GOT and GPT compared with the placebo group have been observed, as well. However, a previous study using another preparation, i.e., curcumin–phosphatidylcholine phytosomes (1000 mg/day), did not reveal any improvement in glycemic indices in individuals with NAFLD following a supplementation period of 8 weeks [24]. This contrast with the results of the present study might be attributed to the potentially greater anti-inflammatory effect (due to phosphatidylserine component) and bioavailability (due to the piperine component) of the phytosomes used in this study compared with the curcumin–phosphatidylcholine phytosomes used in the above-mentioned trial [24].

The positive impact of curcumin per se on FPG is well known and supported by meta-analyses of randomized controlled trials [25]. In our trial, both groups experienced significant improvement in FBG despite changes in insulin resistance parameters. Curcumin improves insulin release while reducing apoptosis and endoplasmic reticulum stress in pancreatic *β* cells, enhances glycolysis and glycogen synthesis while reducing gluconeogenesis in the liver, increases glucose uptake, glycolysis and glycogen synthesis in skeletal muscles, and enhances adiponectin synthesis in the adipose tissue, all leading to improved glycemic control [26]. This suggests that the glucose-lowering activity of the tested phytosomal preparation deserves to be further investigated in diabetic patients, especially considering the promising findings revealing the protective effects of curcumin against macro- and microvascular complications of T2DM [27]. It is worth noting that the previous meta-analysis failed to detect any improvement in HOMA-IR with curcumin supplementation [26] while this parameter was clearly improved in the current trial. Owing to the established link between inflammation and insulin resistance [28], it is suggested that the insulin-sensitizing effect observed in this study could be a reflection of the potent anti-inflammatory activity of the preparation used due to the presence of phosphatidylserine [15].

Another beneficial effect of curcumin that was observed in this study was improvement of hepatic function as reflected by a significant reduction in the activities of GOT, GPT and gGT enzymes as well as FLI score. These improvements are particularly important for the management of NAFLD. This finding is consistent with those of a previous 8-week trial with curcumin–phosphatidylcholine phytosomes (1000 mg/day) showing improvement of hepatic function and steatosis grading in patients with NAFLD [23]. Likewise, another 8-week trial in patients with NAFLD indicated similar findings in terms of hepatic parameters and steatosis severity [29]. Curucmin is known to ameliorate several metabolic risk factors of NAFLD via modulating lipid, glycemic, inflammatory and oxidative pathways [8]. Considering the positive effects observed and the paucity of approved medications for NAFLD, it would be worthwhile to verify the hepatoprotective activity of phosphatidylserine-containing curcumin phytosomes in NAFLD patients.

In this study, we observed a significant effect of curcumin phytosomes in reducing serum TG concentrations. This finding further confirms previous clinical findings suggesting the hypotriglyceridemic activity of curcumin. In particular, a recent meta-analysis of 20 randomized controlled trials revealed a significant TG-lowering activity of curcumin supplementation [7]. Several basic and experimental studies have shown the modulatory action of curcumin on the enzymes and transcription factors involved in both TG biosynthesis and catabolism [30]. Hypertriglyceridemia is associated with several metabolic disorders including metabolic syndrome and NAFLD, and has been suggested to accelerate insulin resistance, especially when it is accompanied by low serum HDL-C concentrations [31]. Hence, the hypotriglyceridemic activity of curcumin could explain, at least in part, the efficacy of this phytochemical in patients with metabolic diseases.

In our study, we found a lowering effect of curcumin supplementation on serum cortisol levels. To our knowledge, this is the first study to report the effect of curcumin on serum cortisol levels in an overweight population with suboptimal FBG. Previous studies in subjects with major depression [32] and in athletes undergoing endurance training [33] did not reveal any alteration of serum cortisol levels following curcumin supplementation. Dysregulated cortisol physiology has been reported in obesity and obesity-related metabolic disorders. Obesity and metabolic syndrome cause hyperactivity of the hypothalamic–pituitary–adrenal axis leading to hypercortisolism, which in turn, contributes to further visceral adipogenesis in a vicious cycle [34, 35]. Several studies have also shown the adverse impact of hypercortisolemia on insulin sensitivity [36, 37], glycemic control [38, 39] and lipid metabolism [40, 41]. Therefore, modulation of serum cortisol levels could be a putative mechanism whereby curcumin can ameliorate metabolic deregulations related to overweight and obesity.

The present study had some limitations that deserve attention. First, this study had a short duration of follow-up and therefore was unable to look at the long-term effects of curcumin supplementation on anthropometric and glycemic indices as well as incidence of metabolic complications such as type 2 T2DM and NAFLD. Second, assessment of the hepatoprotective action of curcumin in this study was based on biochemical assays and it remains to be ascertained if the phytosomal preparation used in this study can favorably affect sonographic and/or histopathological features of NAFLD. Finally, this study assessed a single dose of phytosomal curcumin and the presence of any dose–response association for the observed beneficial effects is open to question.

In conclusion, the present trial indicated that supplementation with a phytosomal preparation of curcumin containing phosphatidylserine and piperine could improve glycemic factors, hepatic function and serum cortisol levels in subjects with overweight and prediabetes. Future studies are warranted to verify the present results in populations with T2DM, metabolic syndrome and NAFLD.
